# ﻿A new species of *Rhacophorus* (Anura, Rhacophoridae) from Guangxi, China

**DOI:** 10.3897/zookeys.1117.85787

**Published:** 2022-08-12

**Authors:** Jing Li, Shuo Liu, Guohua Yu, Tao Sun

**Affiliations:** 1 Key Laboratory of Ecology of Rare and Endangered Species and Environmental Protection (Guangxi Normal University), Ministry of Education, Guilin 541004, China; 2 Guangxi Key Laboratory of Rare and Endangered Animal Ecology, College of Life Science, Guangxi Normal University, Guilin 541004, China; 3 Kunming Natural History Museum of Zoology, Kunming Institute of Zoology, Chinese Academy of Sciences, Kunming 650223, China

**Keywords:** 16S rRNA, morphology, *Rhacophorusnapoensis* sp. nov., *Rhacophorusrhodopus*, taxonomy

## Abstract

Based on morphological and molecular evidence of five male adult specimens collected from Napo County, Baise City, Guangxi Zhuang Autonomous Region, China, we describe a new species of *Rhacophorus*, *Rhacophorusnapoensis***sp. nov.** This new species is similar to *Rhacophorusrhodopus* Liu & Hu, 1959 and *Rhacophorusbipunctatus* Ahl, 1927 in morphology, but it can be distinguished from the latter two by the following morphological characteristics: head width is greater than head length, snout pointed, loreal region oblique, tympanum distinct, maxillary teeth distinct, tongue cordiform, external single subgular vocal sac, tibiotarsal articulation reaches the snout, tibia length is greater than foot length and slightly greater than half of snout-vent length, and single outer metatarsal tubercle is flat. The phylogenetic tree constructed based on 16S rRNA sequence shows that all individuals of this species clustered into the same clade, and genetically this new species differs from *R.rhodopus* and *R.bipunctatus* by 7.71% and 7.98% in 16S rRNA sequences, respectively.

## ﻿Introduction

Currently, the genus *Rhacophorus* Kuhl & Van Hassalt, 1822 contains 43 species (Frost 2021), distributed widely across China, Japan, India, and from the Philippines to Sulawesi (O’Connell et al. 2018). The main common morphological characteristics of *Rhacophorus* are: relatively medium or large body size, intercalary cartilage between terminal and penultimate phalanges of digits present, terminal phalanges of finger and toes Y-shaped, end of the finger expands into a circular disks bearing circum-marginal grooves, webbing exists on all fingers, pupil horizontal, skin is not co-ossified with the skull, extensive dermal folds along the forearm or tarsus present, and dorsal color (usually) brown or green (Li et al. 2012; Pan et al. 2017; Jiang et al. 2019). *Rhacophorusrhodopus* Liu & Hu, 1959 can be distinguished from other members of the genus by its medium-sized and slim body size, well-developed dermal folds along the limbs and a square skin fold dorsal to the anus present, orange brown back with small black spots, a large black spot on the flank, scarlet webbing between the toes, and a golden iris (Fei et al. 2009; Nguyen et al. 2020). *Rhacophorusrhodopus* is mainly distributed in Myanmar (Kachin and Shan states), northeast India, northern Thailand, Laos, Vietnam and southern China (Yunnan, Guangxi and Hainan) (Zug and Kahn 2022).

The classification of *Rhacophorus* has been of concern to many scholars. With the completion of several phylogenetic studies in recent years, the classification of *Rhacophorus* has been clarified, but it has not been completely solved. There are still many disputes. Inger et al. (1999) once thought that *R.rhodopus* and *R.bipunctatus* Ahl, 1927 were the same species, but subsequent studies have shown that they can be distinguished according to the back color, the number of axillary black spots and the color of web. However, the above characteristics of the two species may be unstable with time, region and individual genetic differences, therefore, this conclusion is not completely reliable (Bordoloi et al. 2007; Fei et al. 2009). Although it is difficult to distinguish them completely in morphology, molecular research results show that they are indeed different species. Li et al. (2008, 2012) showed a distant genetic relationship between *R.rhodopus* from Mengyang, Yunnan (type locality) and *R.bipunctatus* from Myanmar (non-type locality), which provides strong evidence to support the view that *R.rhodopus* and *R.bipunctatus* are two different species. In addition, the above research also showed that there are morphological and genetic differences among *R.rhodopus* from different places, and there may be cryptic species within that species. Recent phylogenetic studies showed that *R.rhodopus* is not monophyletic, but belongs to complex (Yu et al. 2007; Tao et al. 2014; Chan et al. 2018). According to these authors and the re-division of *Rhacophorus* by Jiang et al. (2019), the genetic relationship between *R.rhodopus* and *R.bipunctatus* is the closest and they form a main clade with *Rhacophorusnorhayatii* Chan & Grismer, 2010, *Rhacophorusreinwardtii* Schlegel, 1840, *Rhacophorusborneensis* Matsui, Shimada & Sudin, 2013, *Rhacophorushelenae* Rowley, Tran, Hoang & Le, 2012, and *Rhacophoruskio* Ohler & Delorme, 2006.

**Figure 1.**
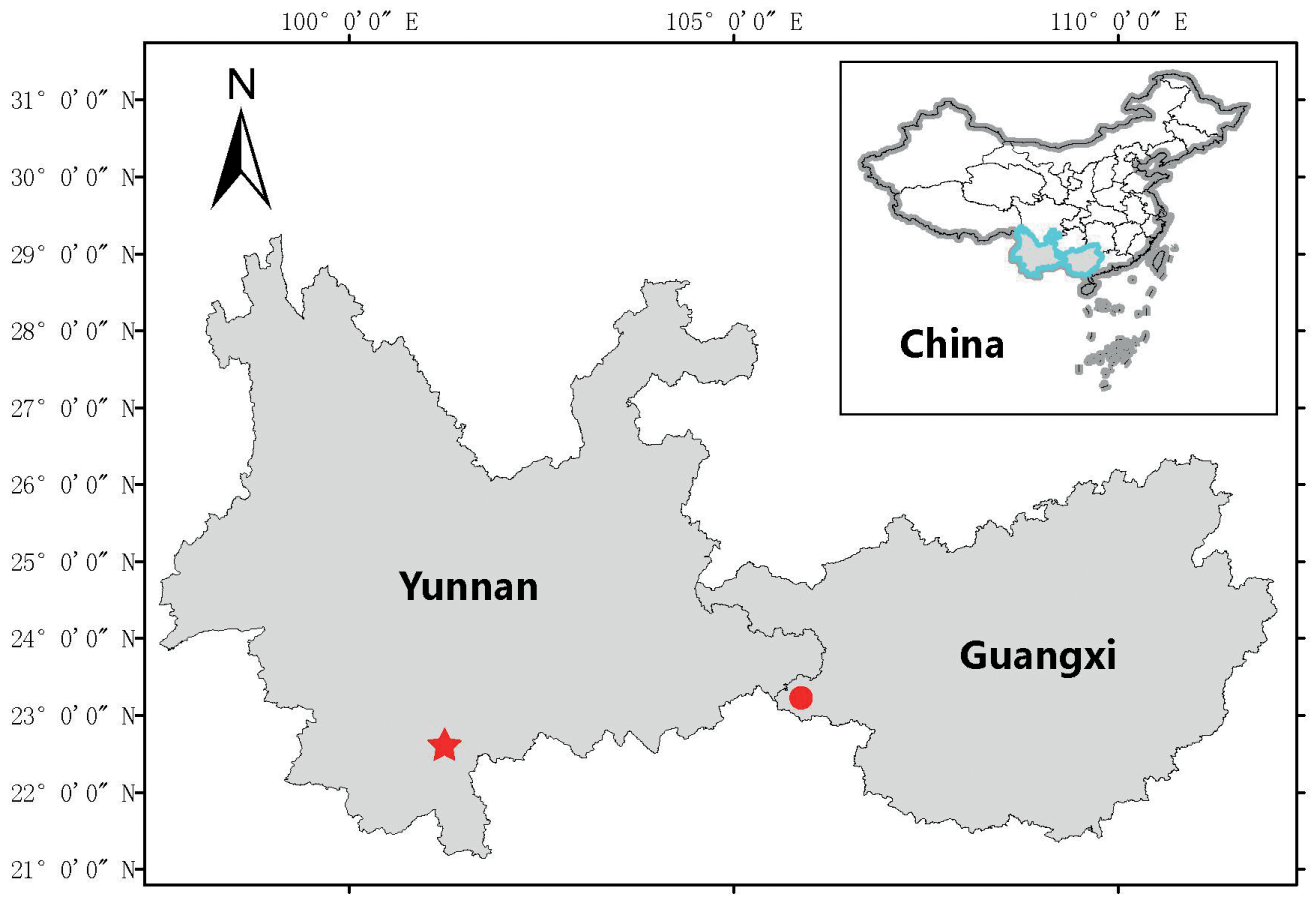
Map shows the collection site of the specimens used in morphological part of this study. The circle represents the type locality of *R.napoensis* sp. nov. at Napo County, Baise City, Guangxi Zhuang Autonomous Region and the star represents the collection site of *R.rhodopus* specimens in the Caiyanghe Nature Reserve, Simao District, Pu’er City, Yunnan Province (the type locality of *R.rhodopus*).

During a field survey in Napo County, Baise City, Guangxi Zhuang Autonomous Region, China (Fig. 1), we collected five specimens of *Rhacophorus* resembling *R.rhodopus* and *R.bipunctatus* in the presence of black spots at axillary region and skin folds above the anus. However, they can be distinguished from *R.rhodopus* and *R.bipunctatus* in morphological and molecular characteristics. Therefore, we consider that these specimens represent a new species of *Rhacophorus*.

## ﻿Materials and methods

### ﻿Sampling

Specimens collected in Napo County, Baise City, Guangxi Zhuang Autonomous Region, China were euthanized and preserved in 75% ethanol. Liver tissue was preserved in 99% ethanol. The specimens are stored in Guangxi Normal University (**GXNU**).

### ﻿Morphology

Morphological data were measured with electronic vernier calipers to the accuracy of 0.1 mm. Morphological terms were referred to Fei (1999). The measured data included the following 16 measurements: snout-vent length (**SVL**, the length from the tip of snout to vent); head length (**HL**, the length from the tip of snout to the posterior edge of the mandibular joint); head width (**HW**, the maximum distance between two sides of the head); snout length (**SL**, the length from the tip of snout to anterior border of eye); internasal space (**INS**, the distance between the inner edges of the left and right nostrils); interorbital space (**IOS**, the narrowest distance between the medial edges of the left and right upper eyelids); width of upper eyelid (**UEW**, the maximum width of upper eyelid); diameter of eye (**ED**, the diameter of the eye parallel to the body axis); diameter of tympanum (**TD**, the maximum diameter of tympanum); distance from nostril to eye (**DNE**, the length from the anterior border of the eye to the inner edge of the ipsilateral nostril); distance from snout to nostril (**SN**, the length from the tip of snout to the inner edge of the ipsilateral nostril); length of forearm and hand (**LAHL**, the length from elbow joint to the tip of the third finger); thigh length (**THL**, the length from vent to knee); tibia length (**TIL**, the length from knee to ankle); length of the foot and tarsus (**TFL**, the length from the tibial tarsal joint to the tip of the fourth toe); foot length (**FL**, the length from the proximal end of the medial metatarsal process to the tip of the fourth toe).

SPSS statistical software was used to analyze 16 morphological measurements of five specimens collected from Napo County, Baise City, Guangxi Zhuang Autonomous Region and four specimens of *R.rhodopus* collected from type locality (Caiyanghe Nature Reserve, Simao District, Pu’er City, Yunnan Province). The snout-vent length data was used to correct the other 15 data measurements (the corrected data was the snout-vent length divided by the quotient of each data). The obtained data were imported into SPSS (statistical product and service solutions) ver. 17.0 for principal component analysis (PCA). The two principal components with the highest contribution rate were used to make a scatter diagram to compare the morphological characteristics between the new species and *R.rhodopus*.

### ﻿Molecular analyses

Total genomic DNA was extracted from liver tissue. Tissue samples were digested with proteinase K, and then purified by standard phenol/chloroform separation and ethanol precipitation. The fragment encoding partial 16S ribosomal RNA (16S) was amplified and sequenced according to the protocol of Yu et al. (2019). All new sequences were deposited in GenBank under accession Nos. ON217794–ON217798.

Homologous sequences of *R.rhodopus*, *R.bipunctatus*, the related species mentioned above, and that for the outgroup, were downloaded from GenBank (Table 1). *Zhangixalussmaragdinus* Blyth, 1852 was selected as outgroup according to Jiang et al. (2019). All sequences were aligned using the default parameters of MUSCLE in MEGA 7 (Kumar et al. 2016). Uncorrected pairwise distances (P-distances) between species were calculated in MEGA 7. The best alternative model was selected as TIMef in MODELTEST ver. 3.7 (Posada and Crandall 1998). Bayesian phylogenetic analysis was conducted using MRBAYES ver. 3.2.6. Two runs were performed simultaneously with four Markov chains starting from a random tree. The chains were run for 5,000,000 generations and sampled every 100 generations. When the average standard deviation of split frequencies was less than 0.01, the first 25% of sampled trees were discarded as burn-in and the remaining trees were used to create a consensus tree and to estimate Bayesian posterior probabilities (BPPs). In addition, we performed maximum likelihood analysis using IQ-TREE2 (Minh et al. 2020) with 1000 bootstrap replicates.

**Table 1.** Species of *Rhacophorus* (and the outgroup, *Zhangixalussmaragdinus*) used in phylogenetic analyses of this study, together with locality and voucher and GenBank accession numbers.

Species	Voucher	Locality	Accession No.
*Z.smaragdinus*	——	——	MN613221
*R.bipunctatus*	PUCZM/IX/SL360	Mizoram, India	MH087073
*R.bipunctatus*	PUCZM/IX/SL612	Mizoram, India	MH087076
*R.helenae*	——	Nui Ong Nature Reserve, Binh Thuan Province, Vietnam	JQ288090
*R.helenae*	——	Nui Ong Nature Reserve, Binh Thuan Province, Vietnam	JQ288091
*R.kio*	SCUM 37941C	Xishuangbanna, Yunnan, China	EU215532
*R.kio*	VN.2018.057	Kon Tum, Vietnam	LC548742
*R.rhodopus*	KIZ589	Longling, Yunnan, China	EF564578
*R.rhodopus*	KIZ587	Longling, Yunnan, China	EF564577
*R.rhodopus*	clone 5	Xishuangbanna, Yunnan, China	EF646366
*R.rhodopus*	SCUM 060692L	Mengyang, Jinghong, China	EU215531
*R.borneensis*	BORN:22410	Sabah, Maliau Basin, Malaysia	AB781693
*R.borneensis*	——	Sabah, Maliau Basin, Malaysia	AB781694
*R.norhayatii*	——	Johor, Endau Rompin, Malaysia	AB728191
*R.reinwardtii*	ENS 16447 (UTA)	Sumatra, Bandung, Indonesia	KY886335
*R.reinwardtii*	ENS 16179 (UTA)	Java, Patuha, Indonesia	KY886328
*R.rhodopus*	L062456	Motuo, Xizang, China	JX219442
*R.rhodopus*	L06245	Motuo, Xizang, China	JX219441
*R.napoensis* **sp. nov.**	GXNU YU000169	Napo, Guangxi, China	ON217794
*R.napoensis* **sp. nov.**	GXNU YU000170	Napo, Guangxi, China	ON217795
*R.napoensis* **sp. nov.**	GXNU YU000171	Napo, Guangxi, China	ON217796
*R.napoensis* **sp. nov.**	GXNU YU000172	Napo, Guangxi, China	ON217797
*R.napoensis* **sp. nov.**	GXNU YU000173	Napo, Guangxi, China	ON217798

## ﻿Results

### ﻿Morphological study

**Figure 2.**
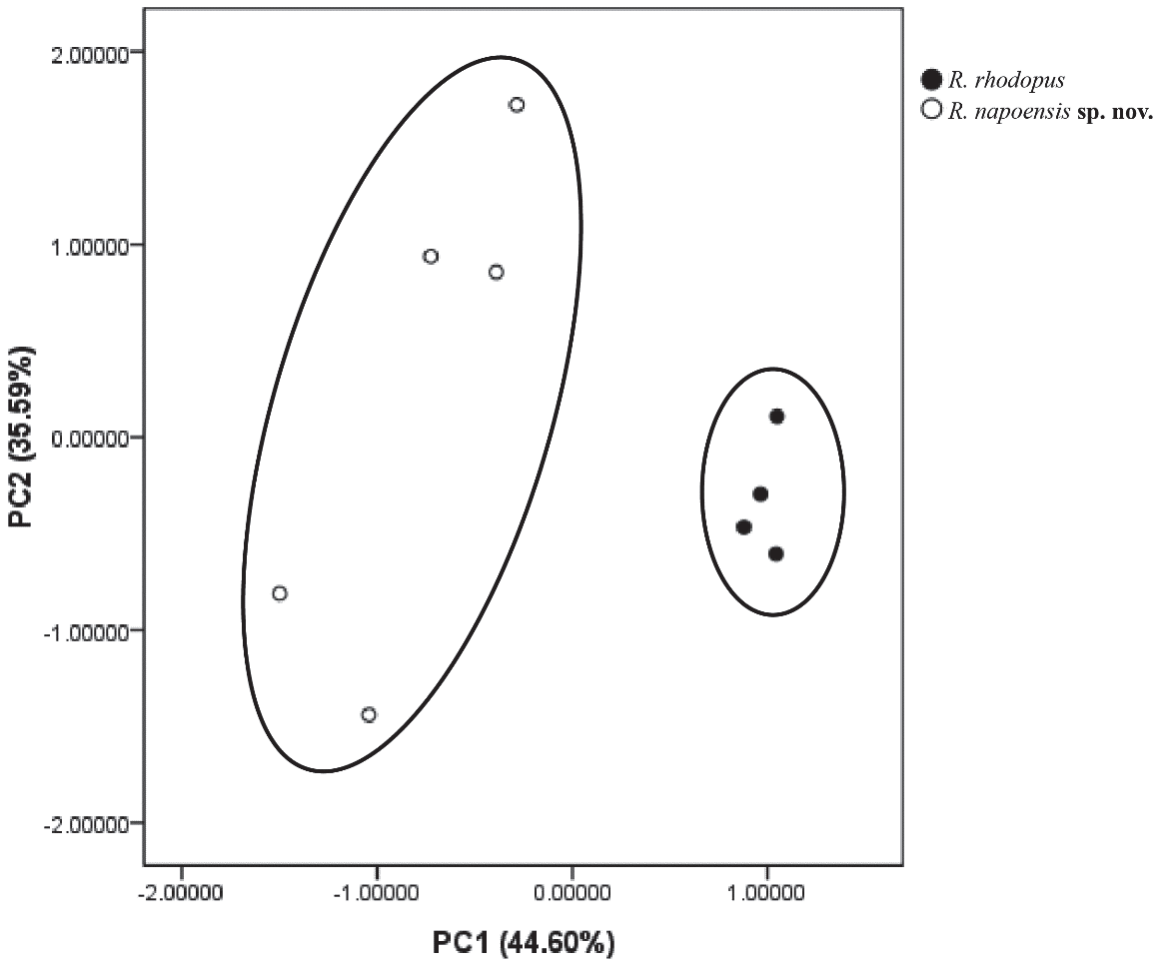
Scatter plot of the principal component analysis of size-adjusted morphological data. The black circles represents *R.rhodopus* from the type locality and the white circles represents *R.napoensis* sp. nov..

Morphological data are summarized in Table 2. We retained the first two principal components which accounted for 80.19% of the total variance (Table 3). Loadings for PC1, which accounted for 44.60% of the total variance, were mainly loaded on head width (HW), snout length (SL), interorbital space (IOS), length of forearm and hand (LAHL), thigh length (THL), tibia length (TIL), length of foot and tarsus (TFL) and foot length (FL). Loadings for PC2, which accounted for 35.59% of the total variance, were mainly loaded on head length (HL), width of upper eyelid (UEW), diameter of eye (ED), diameter of tympanum (TD) and distance from nostril to eye (DNE) (Table 3). The scatter plot based on PC1 and PC2 showed that the new species and *R.rhodopus* can be well distinguished in the X-axis direction, but there is no obvious separation in the Y-axis, indicating that PC1 can be used to distinguish new species from *R.rhodopus* (Fig. 2). The results showed that the snout length of the new species is longer than that of *R.rhodopus*, but the head width, interorbital space, length of forearm and hand, thigh length, tibia length, length of foot and tarsus and foot length are shorter than that of *R.rhodopus*.

**Table 2.** Measurements (mm) and the number of dark spots (left, right, total) at axillary region of *R.napoensis* sp. nov. from Napo County, Baise City, Guangxi Zhuang Autonomous Region, China and *R.rhodopus* from Caiyanghe Nature Reserve, Simao District, Pu’er City, Yunnan Province, China. Abbreviations defined in Materials and methods.

Species	Vouchers	Sex	SVL	HL	HW	SL	INS	IOS	UEW	ED	TD	
*R.napoensis* **sp. nov.**	GXNU YU000169	M	39.6	11.7	14.6	7.3	4.7	5.9	3.7	4.6	2.4	
*R.napoensis* **sp. nov.**	GXNU YU000170	M	43.6	13.5	16.6	8.0	5.1	5.2	4.3	6.0	2.8	
*R.napoensis* **sp. nov.**	GXNU YU000171	M	39.8	16.6	15.5	7.1	5.2	5.0	4.7	6.7	3.7	
*R.napoensis***sp. nov**.	GXNU YU000172	M	38.6	14.7	15.0	6.9	4.9	4.2	4.3	5.8	3.4	
*R.napoensis* **sp. nov.**	GXNU YU000173	M	41.1	15.8	16.3	7.2	5.0	5.3	4.8	6.3	3.6	
*R.rhodopus*	090142	M	35.1	12.7	13.8	5.7	4.4	4.6	3.3	5.1	2.8	
*R.rhodopus*	090143	M	31.4	10.8	12.7	5.1	4.1	4.4	3.1	4.7	2.4	
*R.rhodopus*	090144	M	35.8	11.7	13.9	5.6	4.7	4.8	3.3	5.3	2.6	
*R.rhodopus*	090145	M	31.2	11.7	12.5	5.1	3.7	4.7	2.9	4.2	2.4	
**Species**	**Vouchers**	**Sex**	**DNE**	**SN**	**LAHL**	**THL**	**TIL**	**TFL**	**FL**	**Left**	**Right**	**Total**
*R.napoensis* **sp. nov.**	GXNU YU000169	M	3.1	4.0	20.6	19.5	19.9	28.4	18.7	3	2	5
*R.napoensis* **sp. nov.**	GXNU YU000170	M	3.6	4.1	20.3	21.5	21.7	29.2	18.7	3	2	5
*R.napoensis* **sp. nov.**	GXNU YU000171	M	2.7	4.2	19.4	19.8	20.1	29.2	18.6	2	2	4
*R.napoensis***sp. nov**.	GXNU YU000172	M	2.6	3.8	18.2	18.8	19.6	27.4	17.6	2	3	5
*R.napoensis* **sp. nov.**	GXNU YU000173	M	2.9	3.9	20.1	21.0	20.8	29.3	18.0	2	3	5
*R.rhodopus*	090142	M	2.6	3.1	18.5	18.6	19.0	26.3	17.2	2	1	3
*R.rhodopus*	090143	M	2.1	3.0	16.4	17.1	16.9	23.1	14.6	2	2	4
*R.rhodopus*	090144	M	2.6	3.2	19.1	19.3	18.6	25.9	17.2	1	1	2
*R.rhodopus*	090145	M	2.2	2.6	16.6	16.3	16.7	22.4	15.5	1	1	2

**Table 3.** Factor loadings of the first two principal components of 15 size-adjusted morphometric characters of *R.napoensis* sp. nov. from Napo County and *R.rhodopus* from Caiyanghe Nature Reserve. Absolute values of loading greater than 0.7 in boldface. Abbreviations defined in Materials and methods.

Character	PC1	PC2
HL	0.247	**0.887**
HW	**0.703**	0.458
SL	-**0.957**	0.051
INS	0.574	0.591
IOS	**0.830**	-0.152
UEW	-0.399	**0.887**
ED	0.159	**0.935**
TD	0.158	**0.930**
DNE	-0.530	-**0.771**
SN	-0.562	0.670
LAHL	**0.772**	-0.471
THL	**0.864**	-0.300
TIL	**0.917**	-0.159
TFL	**0.709**	0.234
FL	**0.842**	-0.091
Percentage of variance	44.604	35.588

Additionally, the specimens from Napo can be morphologically distinguished from *R.bipunctatus* by a series of characters: i.e., head width greater than head length, tympanum distinct, and two or three black spots at axillary region.

### ﻿Molecular study

**Figure 3.**
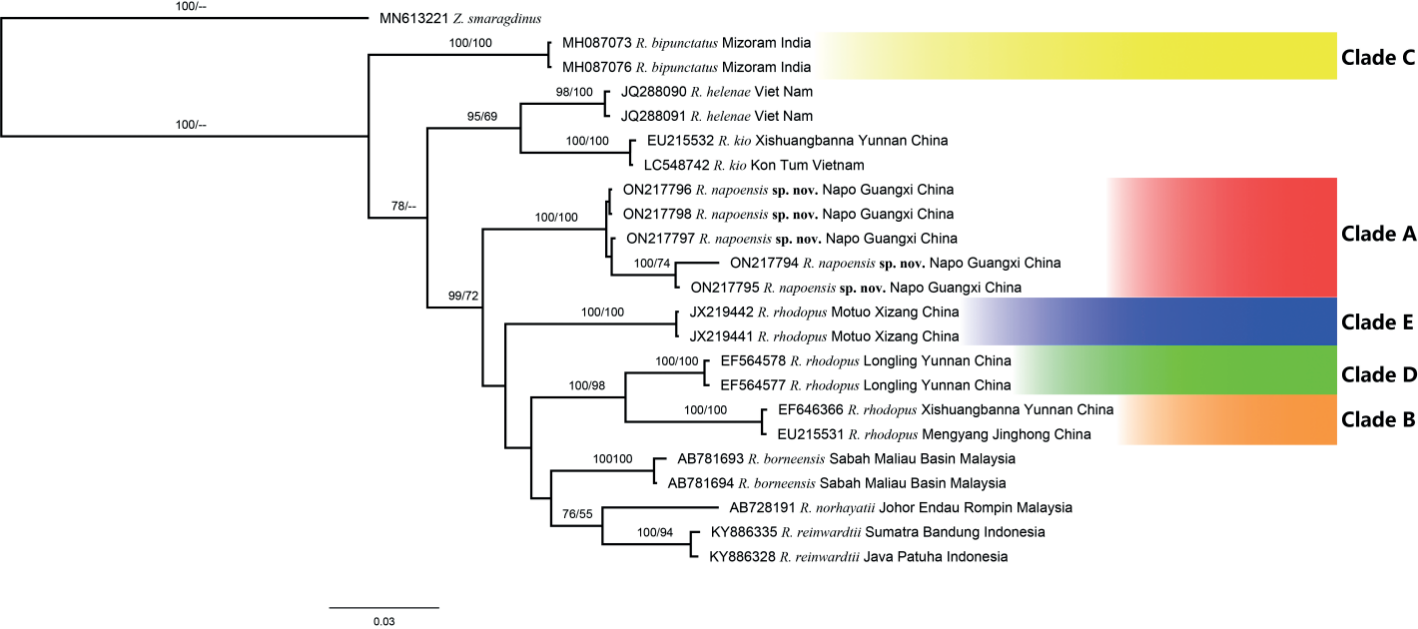
Bayesian phylogenetic tree of *R.rhodopus* and the related species mentioned above constructed with 965bp 16S rRNA gene. The values above the branch are Bayesian posterior probabilities (BPP) and maximum likelihood (ML) bootstrap value, respectively (only values greater than 50% are displayed).

The specimens from Napo County form a clade with strong support (Clade A; Fig. 3). *Rhacophorusrhodopus* from different localities were grouped into three clades, one (Clade B) consisting of samples from the type locality (Xishuangbanna), one (Clade D) consisting of samples from western Yunnan (Longlin), and one (Clade E) containing samples from Motuo, Tibet. The clade consisting of specimens from Napo (Clade A) is not directly related to any one of these clades, although the support values are not strong. The Clade B is sister to the Clade D with strong support. Genetically the clade containing specimens from Napo (Clade A) differs from *R.bipunctatus* (Clade C) and *R.rhodopus* from the type locality (Clade B) by 7.98% and 7.71%, respectively (Table 4). The genetic distance between the specimens from Napo County (Clade A) and *R.rhodopus* from Longling (Clade D) is 6.55%, and the distance between the Napo specimens and *R.rhodopus* from Motuo (Clade E) is 7.59%. All these estimations of genetic distances are greater than 3.0%, the conventional threshold of species-level divergence in 16S rRNA gene of Anura (Vences et al. 2005), meaning that the clade of specimens from Napo is not conspecific with other clades.

**Table 4.** Average uncorrected pairwise distance (%) between groups of 16S rRNA sequences used in this study.

**Species**	**1**	**2**	**3**	**4**	**5**	**6**	**7**	**8**	**9**	**10**
1 *R.napoensis***sp. nov.** (Clade A)										
2 *R.rhodopus* (Clade D)	6.55									
3 *R.rhodopus* (Clade B)	7.71	4.84								
4 *R.rhodopus* (Clade E)	7.59	5.97	7.69							
5 *R.bipunctatus* (Clade C)	7.98	7.26	8.73	9.14						
6 *Z.smaragdinus*	15.28	11.34	13.79	15.60	14.35					
7 *R.borneensis*	6.55	5.86	8.06	6.54	9.06	14.99				
8 *R.helenae*	6.67	7.20	9.47	6.65	7.78	9.68	5.30			
9 *R.kio*	8.50	7.96	9.67	7.78	8.82	15.22	7.60	4.60		
10 *R.norhayatii*	7.83	5.76	7.26	7.94	9.59	16.49	5.47	7.39	9.03	
11 *R.reinwardtii*	4.64	5.74	6.49	5.00	6.79	11.03	4.89	6.34	7.67	4.04

Therefore, based on the above morphological and molecular evidence, we considered that the specimens from Napo County (Clade A) are different from *R.rhodopus* (Clades B, D and E) and *R.bipunctatus* (Clade C) and represent a new species of *Rhacophorus*, which is described in the Taxonomy section below. As for Clades D and E, we suppose that they likely represent two cryptic species confused with *R.rhodopus*, pending further morphological studies.

### ﻿Taxonomy

#### ﻿ Rhacophorus napoensis sp. nov.

Taxon classificationAnimaliaAnuraRhacophoridae

C1D0BC5B-96C9-5889-8B44-C37AAFCD3449

https://zoobank.org/66C47824-DE9B-4EA7-AFF1-CFBA0F8D239E

Figs 4 , 5 , 6

##### Material examined.

***Holotype*.**GXNU YU000172, adult male, collected on 25 March 2019 by Shuo Liu from Napo County, Baise City, Guangxi Zhuang Autonomous Region, China (23°1'20"N, 105°50'58"E, ca 1032 m a.s.l.). ***Paratypes*.**GXNU YU000169, GXNU YU000170, GXNU YU000171 and GXNU YU000173, four adult males, collected at the same time as the holotype from the type locality by Shuo Liu.

##### Etymology.

The specific epithet is named for the type locality. We suggest the English common name as “Napo tree frog” and the Chinese common name as “那坡树蛙”.

##### Diagnosis.

Morphologically, there are the following differences between Napo County specimens and other species belonging to *Rhacophorus*: (1) Medium body size (adult males SVL 38.6–43.6 mm); (2) snout pointed, projecting beyond margin of lower jaw in ventral view, and the tip has a distinct bulge; (3) tympanum distinct, rounded; (4) maxillary teeth distinct; (5) tongue cordiform, notably notched posteriorly; (6) external single subgular vocal sac; (7) the tibiotarsal articulation reaches the snout; (8) TIL longer than FL and slightly longer than half of SVL; (9) entire web between fingers and toes; (10) single inner metatarsal tubercle, flat; (11) banding exists in dorsal surface of limbs posterior part of dorsum; (12) two to three black spots at axillary region; (13) web is not black; and (14) dorsal color hoary with numerous black spots when the species is kept in preservative.

##### Description of holotype.

Adult male, body size medium (SVL 38.6 mm); head width (HW 15.0 mm) longer than head length (HL 14.7 mm); snout pointed, longer than diameter of eye (ED 5.8 mm), protruding from the margin of the lower jaw; canthus rostralis distinct; loreal region oblique; nostril small, closer to eye than to tip of snout; internasal space (INS 4.9 mm) longer than interorbital space (IOS 4.2 mm) and width of upper eyelid (UEW 4.3 mm); interorbital space (IOS 4.2 mm) almost equal to width of upper eyelid (UEW 4.3 mm); pineal ocellus absent; pupil horizontal; tympanum distinct, rounded, diameter of tympanum (TD 3.4 mm) longer than half eye diameter (ED 5.8 mm), internasal space (INS 4.9 mm) and half interorbital space (IOS 4.2 mm); supratympanic fold distinct; vomerine teeth present; maxillary teeth distinct; tongue cordiform, attached anteriorly, notably notched posteriorly; choanae oval; external single subgular vocal sac, vocal sac opening at the bottom of the mouth on either side.

**Figure 4.**
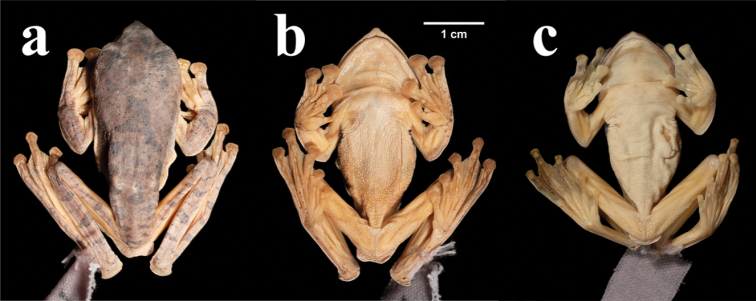
Dorsal views (**a**) and ventral views (**b**) of the holotype of *R.napoensis* sp. nov. (GXNU YU000172) in preservative. Ventral view (**c**) of *R.rhodopus* from type locality (090142) in preservative.

**Figure 5.**
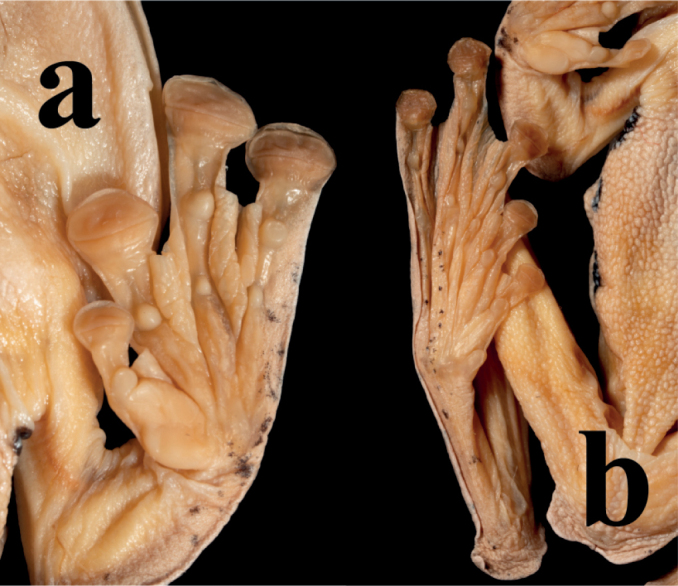
Ventral view of hand (**a**) and foot (**b**) of the holotype of *R.napoensis* sp. nov. (GXNU YU000172) in preservative.

Forelimbs stubby, length of lower arm and hand (LAHL 18.2 mm) shorter than snout-vent length (SVL 38.6 mm); fingers short, relative length of fingers: I < II < IV < III; tips of all fingers expanded into discs; entire web between fingers; subarticular tubercles prominent and rounded, formula 1, 1, 2, 2; supernumerary tubercles below the base of finger absent; single thenar (inner metacarpal) tubercle large, oval, distinct (Fig. 5a).

Hindlimbs long and thin, tibia length (TIL 19.6 mm) longer than thigh length (THL 18.8 mm) and foot length (FL 17.6 mm), tibiotarsal articulation reaches the snout; when the legs are at right angles to the body, the heels overlap; relative length of toes is I < II < III < V < IV; tips of all toes expanded into discs; entire web between toes; subarticular tubercles prominent and rounded, formula 1, 1, 2, 3, 2; supernumerary tubercle below the base of toe absent; single inner metatarsal tubercle, flat (Fig. 5b).

**Figure 6.**
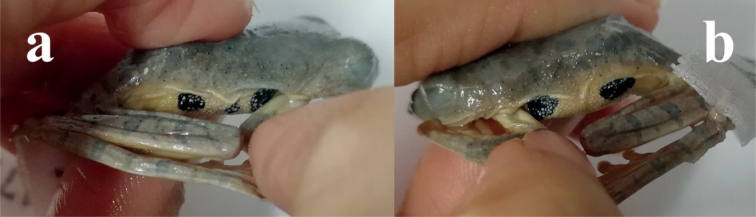
Right armpit and flank view (**a**) and the left armpit and flank view (**b**) of the holotype of *R.napoensis* sp. nov. (GXNU YU000172) in preservative.

The skin of throat, ventral part of tibia, foot and tarsus smooth; the skin of chest, venter, vent and thigh rough and granular; some warts are found around the vent and flanks; dermal fringe along joint, vent and the outer sides of limbs (Fig. 4b); three black spots at the right armpit (Fig. 6a), and two black spots at the left armpit (Fig. 6b).

***Color of holotype in preservative*.** Dorsal color hoary with numerous black spots; horizontal banding on dorsum and dorsal surface of limbs (Fig. 4a).

***Male secondary sexual characteristics*.** No nuptial pad and lineae masculinae were observed.

##### Morphological variation.

The morphological measurement and the number of dark spots at axillary region of the holotype and paratypes are shown in Table 2. The total number of dark spots at axillary region varies between individuals, and the number of dark spots on the left and right axillary region also varies. Because all specimens are male, sexual dimorphism cannot be determined.

##### Distribution and ecology.

**Figure 7.**
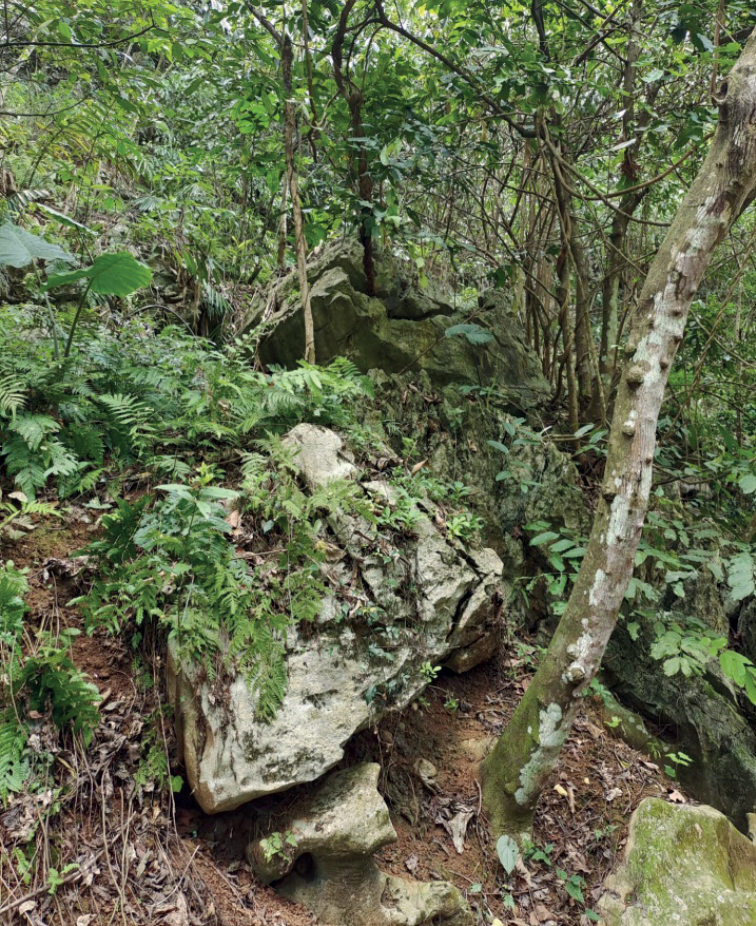
Habitat of *R.napoensis* sp. nov. at the type locality.

The new species was found near several large rocks in the bushes, 306 m southeast of Nongyao, Napo County, Baise City, Guangxi Zhuang Autonomous Region, China (Fig. 7). Vocal recordings and tadpoles of this new species were not collected.

##### Comparisons.

The new species is obviously distinguishable from most of the closely-related species including *R.norhayatii*, *R.reinwardtii*, *R.kio*, *R.borneensis*, and *R.helenae* by smaller body size (SVL of adult males 38.6‒43.6 mm vs. 64.7 mm in *R.norhayatii*, 41.1‒52.5 mm in *R.reinwardtii*, 70.5 mm in *R.kio*, 50.9 mm in *R.borneensis*, and 72.3‒85.5 mm in *R.helenae*) and lack of black coloration on the webs (vs. webs between toes black).

The new species differs from *R.rhodopus* by head width greater than head length (vs. head length almost equal to head width), snout pointed and the tip has a distinct bulge (vs. snout oblique and pointed) (Fig. 4), tongue cordiform, notably notched posteriorly (vs. tongue narrow and long, deeply notched posteriorly), external single subgular vocal sac (vs. internal single subgular vocal sac), the tibiotarsal articulation reaches the snout (vs. the tibiotarsal articulation reaches the eye), tibia length is slightly greater than half of snout-vent length (vs. tibia length is about half of snout-vent length), two to three black spots at axillary region (vs. one black or dark round spot at axillary region); and from *R.bipunctatus* by head width greater than head length (vs. head length almost equal to head width), snout pointed, and the tip has a distinct bulge (vs. snout broad and pointed), loreal region oblique (vs. loreal region concave), tympanum distinct (vs. tympanum indistinct), maxillary teeth distinct (vs. tooth-like projection on maxilla absent), tongue cordiform, notably notched posteriorly (vs. tongue medium size, round, slight notched posteriorly, median lingual process absent), slender toes (vs. toes rather short, thin), tibia length is slightly greater than half of snout-vent length (vs. tibia length is slightly less than half of snout-vent length), two to three black spots at axillary region (vs. one big and one small black spot at axillary region).

## ﻿Discussion

The phenomenon of cryptic species was first discovered by Derham in 1718 (Winker 2005), which refers to species that are highly similar in morphology and are hidden as known species (Lincoln et al. 1983). Cryptic species and known species form a species complex (Liu 2012). The phylogenetic tree constructed in this study shows that *R.rhodopus* is clustered into three clades (Clades B, D, and E) and the new species from Napo (Clade A) is not directly related to these clades or *R.bipunctatus* (Clade C) with strong supports. Furthermore, genetically the new species differs from Clades B‒E by over 6.5%, which is greater than the conventional threshold of species-level divergence in 16S rRNA sequence of Anura (3%; Vences et al. 2005), and morphologically the new species can be distinguished from *R.rhodopus* and *R.bipunctatus* by a series of characters (i.e., longer snout, shorter tibia length, and head width greater than head length). Therefore, we think that *Rhacophorusnapoensis* sp. nov. should be diagnosed as an independent species.

Genetically, the clade containing specimens from the type locality of *R.rhodopus* (Clade B) differs from specimens from Longling, Yunnan, China (Clade D) and specimens from Motuo, Tibet, China (Clade E) by 4.84% and 7.69%, and the differences were greater than 3%, which means that probably the Clades D and E are not *R.rhodopus*, but two separate species (Vences et al. 2005; Vieites et al. 2009), which are in line with the concept of cryptic species. A morphological study is necessary to confirm the taxonomic status of the Clades D and E. This result supports that there may be cryptic species in *R.rhodopus* and that *R.rhodopus* belongs to complex. Species are the basic units of biological taxonomy. Only by making a clear and scientific distinction between species can we further study the interspecific relationship, biodiversity and evolution of species. With the maturity of research technology, especially the rapid development of molecular systematics, more and more species complexes have been deeply studied (Villalobos-Guerrero 2019; Amorim et al. 2022; Guimarães et al. 2022;), which shows that there may be some mistakes in the current species classification, and there are more cryptic species waiting to be discovered. The study of cryptic species and complexes will have a far-reaching impact on evolutionary theory, biogeography and conservation planning, which needs attention (Bickford et al. 2007).

Recently, Poyarkov et al. (2021) mentioned that the existing records of *R.bipunctatus* in Thailand, Laos, Cambodia and Vietnam seem to be related to *R.rhodopus*, and the classification of this complex needs to be further clarified. These countries are located in the Indochina Peninsula, bordering on the southwest of China. The terrain of this area is mainly mountainous and plateau (Qiu et al. 2021). The complex landform may lead to geographical isolation within the widely distributed species, thus forming new species (Wu et al. 2020). Napo County, Baise City, Guangxi Zhuang Autonomous Region, where the new species was collected, and the type locality of *R.rhodopus*, are located in this area. Therefore, we speculate that there may be more undiscovered new species in the Indochina Peninsula and its surrounding areas. In conclusion, the intraspecific classification of *R.rhodopus* and the morphological differences between this species and *R.bipunctatus* are still vague. There may be taxonomic disputes and errors among different geographical populations of *R.rhodopus*. The intraspecific morphological differences and phylogenetic relationship of *R.rhodopus* need to be further studied.

## Supplementary Material

XML Treatment for Rhacophorus napoensis
